# Production and Characterization of Chitooligosaccharides: Evaluation of Acute Toxicity, Healing, and Anti-Inflammatory Actions

**DOI:** 10.3390/ijms221910631

**Published:** 2021-09-30

**Authors:** Rafael Caetano Lisbôa Castro de Andrade, Nathália Kelly de Araújo, Manoela Torres-Rêgo, Allanny Alves Furtado, Alessandra Daniele-Silva, Weslley de Souza Paiva, Julia Maria de Medeiros Dantas, Nayara Sousa da Silva, Arnóbio Antônio da Silva-Júnior, Marcela Abbott Galvão Ururahy, Cristiane Fernandes de Assis, Leandro De Santis Ferreira, Hugo Alexandre Oliveira Rocha, Matheus de Freitas Fernandes-Pedrosa

**Affiliations:** 1 Laboratory of Technology and Pharmaceutical Biotechnology (Tecbiofar), College of Pharmacy, Federal University of Rio Grande do Norte, Natal 59012-570, Brazil; 1995rafaelcastro@gmail.com (R.C.L.C.d.A.); nakar_rn@hotmail.com (N.K.d.A.); allannyfurtado@ufrn.edu.br (A.A.F.); alessandradaniele@ufrn.edu.br (A.D.-S.); nay.sou@hotmail.com (N.S.d.S.); arnobiosilva@gmail.com (A.A.d.S.-J.); 2 Graduate Program of Chemistry, Chemistry Institute, Federal University of Rio Grande do Norte, Natal 59072-970, Brazil; 3 Laboratory of Biotechnology of Natural Biopolymers, Department of Biochemistry, Bioscience Center, Federal University of Rio Grande do Norte, Natal 59072-970, Brazil; wdspaiva@gmail.com (W.d.S.P.); hugo-alexandre@uol.com.br (H.A.O.R.); 4 Postgraduate Program in Chemical Engineering, Technology Center, Federal University of Rio Grande do Norte, Natal 59072-970, Brazil; medeirosjuulia@gmail.com; 5 Department of Clinical Analysis and Toxicology, College of Pharmacy, Federal University of Rio Grande do Norte, Natal 59012-570, Brazil; marcelaururahy@yahoo.com.br (M.A.G.U.); cristianeassis@hotmail.com (C.F.d.A.); 6 Department of Pharmacy, College of Pharmacy, Federal University of Rio Grande do Norte, Natal 59012-570, Brazil; lean_sf@yahoo.com.br

**Keywords:** biocompatibility, oligosaccharides, chitosan, enzymatic hydrolysis, in vivo tests

## Abstract

The search for promising biomolecules such as chitooligosaccharides (COS) has increased due to the need for healing products that act efficiently, avoiding complications resulting from exacerbated inflammation. Therefore, this study aimed to produce COS in two stages of hydrolysis using chitosanases derived from *Bacillus toyonensis.* Additionally, this study aimed to structurally characterize the COS via mass spectrometry, to analyze their biocompatibility in acute toxicity models in vivo, to evaluate their healing action in a cell migration model in vitro, to analyze the anti-inflammatory activity in in vivo models of xylol-induced ear edema and zymosan-induced air pouch, and to assess the wound repair action in vivo. The structural characterization process pointed out the presence of hexamers. The in vitro and in vivo biocompatibility of COS was reaffirmed. The COS stimulated the fibroblast migration. In the in vivo inflammatory assays, COS showed an antiedematogenic response and significant reductions in leukocyte migration, cytokine release, and protein exudate. The COS healing effect in vivo was confirmed by the significant wound reduction after seven days of the experiment. These results indicated that the presence of hexamers influences the COS biological properties, which have potential uses in the pharmaceutical field due to their healing and anti-inflammatory action.

## 1. Introduction

Chitin, in addition to being a valuable component in arthropod exoskeletons, is also present in fungal cell walls and can be used as a raw material to obtain other substances [1]. For this, it is necessary to convert this molecule into certain derivatives, which are of great scientific interest in relation to their effects in several fields of research [2]. In view of this, the chitin deacetylation process makes it possible to obtain chitosan, a multifunctional polysaccharide that has been widely researched in the cosmetology, medical, chemical, and pharmaceutical fields [3,4]. Chitosan has antioxidant, antimicrobial, and anti-inflammatory actions, as highlighted in the literature, and stands out due to its absence of toxicity and for being biodegradable and biocompatible [5,6,7,8,9,10]; however, because this polysaccharide has a high molecular weight, high viscosity at neutral pH, and is insoluble in water, its use in biological systems is limited [11,12]. As such, the strategy of hydrolyzing this polymer has been used to decrease its viscosity and enhance its biological effects [2].

Oligomers originated by hydrolysis are called chitooligosaccharides (COS). Due to their non-toxic character and solubility in water, their applicability has been investigated in different areas, such as in agriculture, medicine, and pharmacy [13,14]. In addition, studies indicate that COS have multiple biological properties, such as anti-inflammatory, immunostimulant, antimicrobial, antitumor, antidiabetic, antioxidant, and neuroprotective effects [15,16,17,18,19,20,21,22,23]. With regard to their healing activity, different in vitro and in vivo studies demonstrate the potential of COS; however, most of these reports consist of the evaluation of these substances together with other products and few have described their action in animal healing trials [24,25]. As such, further studies are needed to better elucidate their effects when repairing skin wounds.

The skin’s functions include providing a barrier that is essential for the survival of all species, so a rupture in this organ, especially in bedridden patients with diabetes or burns, is very alarming, as the tissue repair process may be compromised [26,27,28]. When this type of injury occurs, the main biological objective is wound repair in order to develop mechanisms that can induce more efficient healing, with participation from immune cells during the process being extremely important and contributing to the release of cytokines and chemokines responsible for stimulating collagen synthesis and the action of fibroblasts [2,29].

The healing action of COS is the result of their proprieties, including their anti-inflammatory, antioxidant, immunostimulatory, antibacterial, and antifungal activities. Moreover, the wound healing process can be accelerated via higher proliferation of fibroblasts and collagenase activity. Some studies involving COS in biomaterial engineering highlight that the healing activity is also mediated by activation of the transforming growth factor-beta (TGF-β)-1-Smad2/3 pathway. This pathway acts in the first stage of wound healing, causing activation of other components, such as transforming growth factor-beta (TGF-β1) and its receptors, collagen I and collagen III [25]. The upregulation of miR-27a (a microRNA related to proliferation and growth signaling pathways) could also be related to the healing capacity of COS [30].

The inflammatory process that occurs at this time is crucial for tissue restoration; however, it can become exacerbated, resulting in chronic wound development [31]. Inflammation is an immune system reaction to chemical, physical, or biological stimuli. The response generated by these stimuli is characterized by vasodilation with plasma protein extravasation, leukocyte migration, and activation of various inflammatory mediators, such as nitric oxide (NO), prostaglandins, and cytokines (IL-1β, IL-6, IL-12, and TNF-α). These and other factors result in symptoms such as pain, erythema, heat, edema, and loss of function [17]. The inflammatory process treatment has the objective of eliminating the symptoms, then non-steroidal anti-inflammatory drugs (NSAIDs) and corticosteroids are used; however, they can cause adverse reactions, leading to cardiovascular, gastric, and renal complications [32,33].

Faced with the need for therapeutic alternatives that have less adverse effects and with efficient anti-inflammatory and healing actions that accelerate the tissue repair process, especially in chronic wound cases, the present study aims to produce chitooligosaccharides via enzymatic hydrolysis using *Bacillus toyonensis* chitosanases; to structurally characterize the products obtained; and to evaluate the acute toxicity in mice, the anti-inflammatory action in vivo, and the healing effect in in vitro and in vivo models. As the anti-inflammatory and healing actions of COS produced through chitosanases obtained from *Bacillus toyonensis* have still not been reported in the literature, the results found here will contribute to demonstrating the potential of these molecules, highlighting the importance of using residues rich in chitin from crustaceans as sources of bioactive components in the pharmaceutical field.

## 2. Results and Discussion

### 2.1. Chitooligosaccharide Production and Characterization

Initially, the bacterium *Bacillus toyonensis* was cultivated to obtain chitosanases. The chitosan hydrolysis occurred twice, at 1 and 5 min, generating samples named COS1 and COS5. The enzyme broth showed enzyme activity of 0.87 U/mL and a protein concentration of 1.05 mg/mL, with a specific activity of 0.82 U/mg. The sample yields obtained were 74% and 62.6% for COS1 and COS5, respectively.

**Figure 1:**
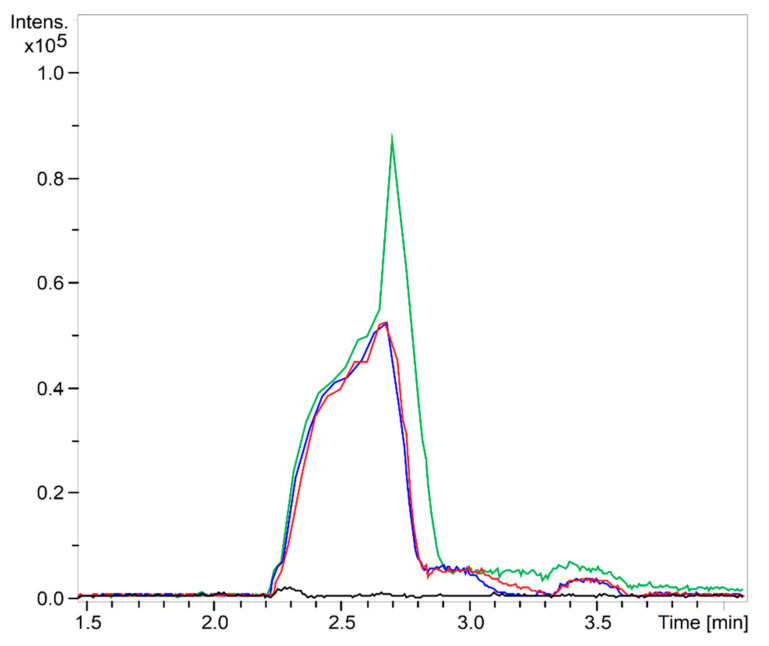
LC-MS/MS chromatograms (ESI-QTOF) obtained for the COS1 sample with an emphasis between 1.5 and 4 min. Black: chitosan; blue: COS1F1; red: COS1F2; green: COS1F3.

**Figure 2:**
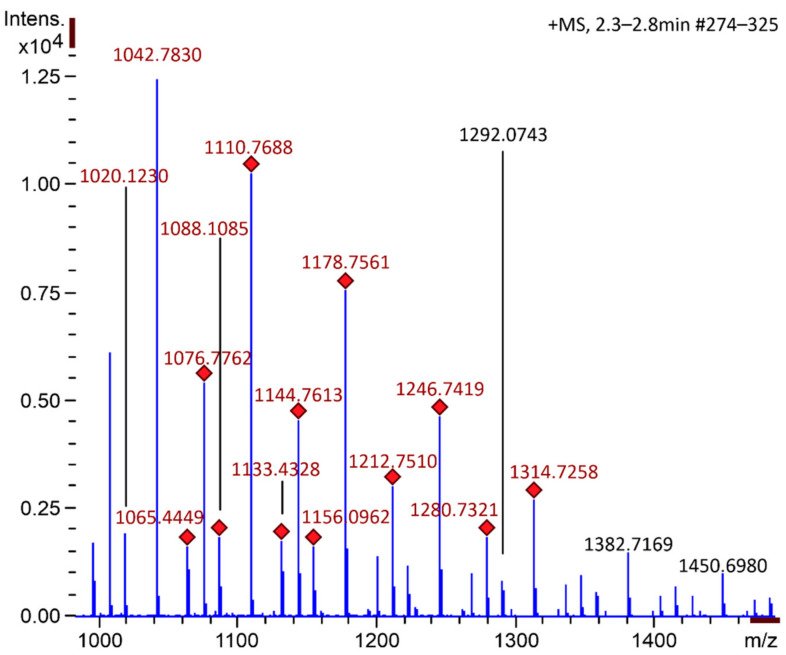
Mass spectrum with an emphasis on the *m/z* region between 950 and 1500 obtained for the peak present in fraction G1 of sample COS1 at the retention times of 2.3 to 2.8 min with subtraction of the baseline.

**Figure 3:**
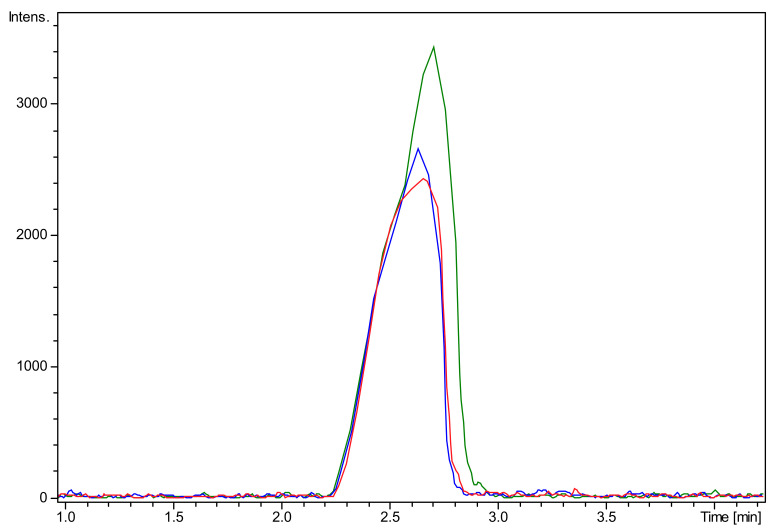
LC-MS/MS chromatograms (ESI-QTOF) obtained for the ion *m/z* 1133 present in the sample COS1 fractions. Blue: COS1G1; red COS1G2; and green COS1G3.

The mass spectrometry data highlighted the efficiency of the method used to obtain COS. It was observed that after 1 min of hydrolysis it was possible to notice the presence of enzymatic degradation products of chitosan (Figure 1). The chromatogram demonstrates for different COS1 fractions (COS1F1, COS1F2, and COS1F3) the presence of peaks of the reaction products that are absent for the chitosan pattern. The mass spectra (Figure 2) of the first peak, with a retention time of 2.3 to 2.8 min, showed several ions, highlighting ion with *m/z* 1133 present in all fractions, as shown in Figure 3. 

Because it has a mass similar to a hexamer and three acetylated groups, the ion *m/z* 1133 was identified as such; these data corroborate the previous results that evidenced the presence of ion *m/z* 1133 in the COS fractions produced with 10 min hydrolysis [18]. Regarding COS5, the structural analysis was carried out in parallel by our research group using the MALDI-TOF/TOF mass spectrometry methodology, which revealed the presence of oligomers with 6 and 7 monomeric units [34]. 

The presence of hexamers and heptamers in the samples is related to the biological properties of COS [35]. Rafael et al. [36] reported that COS composed of a mixture of monomers, hexamers, and heptamers strongly inhibited the growth of antibiotic-resistant Gram-positive and Gram-negative bacteria; thus, the presence of these oligomers in COS1 and COS5 influenced the therapeutic effects of these samples.

### 2.2. Cell Viability

**Figure 4:**
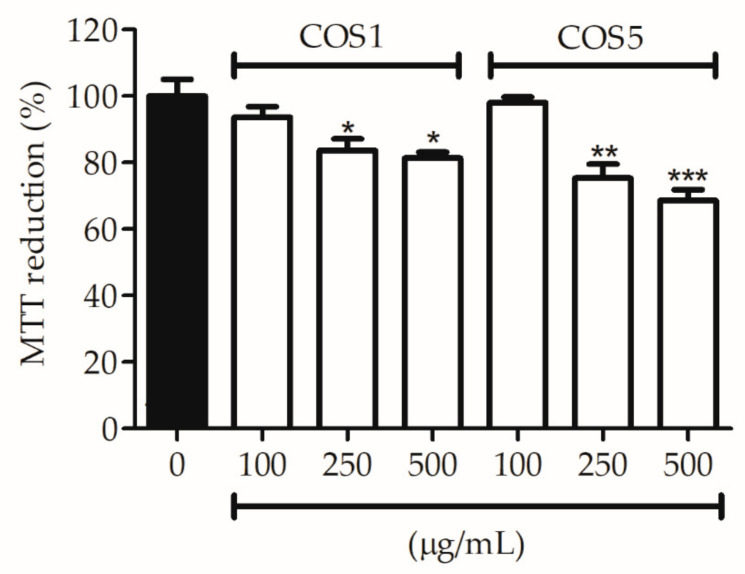
Cell viability of 3T3 fibroblasts incubated with COS1 and COS5 at different concentrations after 24 h of incubation. Note: * p < 0.05, ** p < 0.003, and *** p < 0.001 compared to the control group (bar black).

As shown in Figure 4, COS1 and COS5 revealed no cytotoxic effects on 3T3 cells at a concentration of 100 µg/mL after 24 h of incubation. At concentrations of 250 and 500 µg/mL, COS1 decreased the cell viability by 16.3% and 18.7%, respectively, while COS5 reduced the cell viability by 24.7% and 31.3% at the same concentrations (Figure 4). This inhibition of the capacity to reduce MTT may be related to the presence, even in a small amount, of glucosamine in the samples. In the literature, Assis et al. (2012) demonstrated glucosamine’s toxicity on murine fibroblasts 3T3; however, in that same study, the oligomers of 5 monomer units did not show cytotoxicity up to a concentration 1 mg/mL, as was found for the concentration of 100 µg/mL for COS1 and COS5. In addition, the COS biocompatibility of in vitro toxicity models containing human dermal fibroblasts (FDHs), mast cells, and more recently murine macrophages and primate epithelial cells has been reported in the literature [18,37,38].

### 2.3. Healing Test (“Scratch”) In Vitro

**Figure 5:**
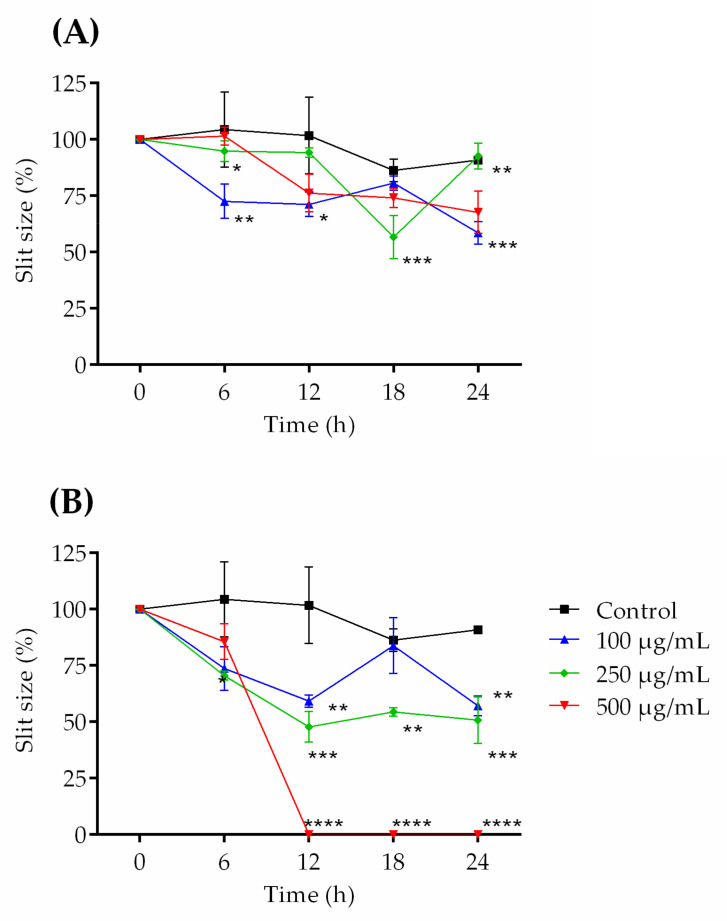
Effects of COS1 (**A**) and COS5 (**B**) on cell migration of murine 3T3 fibroblast cells. The results are expressed as percentages of the slit size. Note: * p < 0.05, ** p < 0.003, *** p < 0.001, and **** p < 0.0001 compared to the control group.

**Figure 6:**
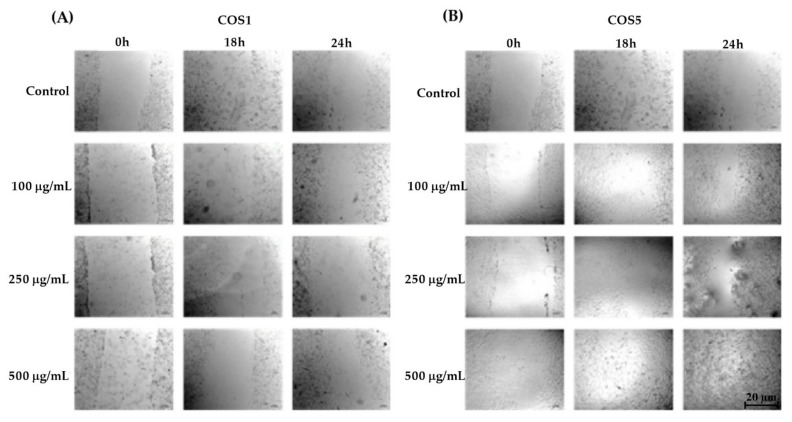
Actions of COS1 (**A**) and COS5 (**B**) at different concentrations in the “scratch” cell migration model demonstrated in the well photos for different groups at 0, 18, and 24 h of the experiment.

The scratch model, characterized by a continuous incision in the confluent cell monolayer, allows the cell migration present at the edge of the scratch to be measured. The greater the cell migration, the smaller the crack size will be, since the migration occurs to repair the lesion. The results demonstrated that the cells incubated with COS1 (Figure 5A and Figure 6A) presented increased cell migration in the first 6 h at the concentration of 100 µg/mL. After 24 h, COS1 (100 and 500 µg/mL) revealed the greatest reduction in wound size in comparison to the control. Regarding COS5 (Figure 5B and Figure 6B), this chitooligosaccharide showed modest reductions of the crack at 100 and 250 µg/mL after 6 h; however, all concentrations induced significant cell migration after 12 h, with continuous effects for the concentrations of 250 and 500 µg/mL until 24 h. The concentration of 100 µg/mL showed an increase in the wound after 18 h, which returned to induce important cell growth at 24 h.

For COS1 250 µg/mL, it is possible to observe an increase in the size of the groove after 18 h of incubation (Figure 5A). The low induction or expression of molecules in the extracellular matrix after incubation with COS1 at this concentration should promote the detachment of weakly adhered cells after 24 h. In addition, cell proliferation may have promoted nutrient depletion from the culture medium, as well as its acidification, and may consequently have favored cell detachment (18 h after slit induction), with proliferation returning after 6 h of incubation (24 h after slit induction).

The concentration of 500 µg/mL COS5 had a strong influence on the wound size. The cell culture that received this concentration after 12 h of the experiment showed total wound closure, making it impossible to visualize any trace of the previously formed fissure (Figure 6B); thus, it was noted that the reduction in the wound size may be related to the concentration, with greater cell migration being observed at higher concentrations of COS1 and COS5.

Although no studies in the literature demonstrate the proliferative effect of 3T3 fibroblasts in the presence of COS produced with chitosanases from *Baccilus toyonensis*, this result demonstrates for the first time the potential of these substances to induce the migration of these cells. This is important, because fibroblasts are essential for efficient tissue repair, being present from the end of the inflammatory stage to the end of the lesion reepithelization process, acting through cytokine release, collagen, growth factors and other components of the extracellular matrix [16].

### 2.4. Acute Toxicity Model

In the acute toxicity test, the 2000 mg/kg doses (limit established by the OECD in 2001) of COS1 and COS5, which were administered intragastrically, did not cause behavioral, neurological, or mortality changes during the observation period (14 days). In the evaluation of animal weight gain and organ relative weight, there was no statistical difference between the groups treated with the COS and phosphate-buffered saline (PBS) (Table 1). There was only a slight increase in the lung weight of the animals that were treated with COS5, which proved not to interfere with the hematological and biochemical findings.

The liver enzymes alanine aminotransferase (ALT) and aspartate aminotransferase (AST), biomarkers that indicate liver damage when they are altered, did not show changes in serum levels in the analysis [39]. Creatinine, which is used as a parameter for kidney injury, also did not show significant changes between groups [40]. Regarding the other biochemical parameters (albumin, glucose, cholesterol, urea, and uric acid), no significant differences were observed; however, there was only an increase in total proteins in the COS5 group when compared to the PBS control (Table 2).

Regarding hematological parameters, statistical changes were observed in terms of the erythrocyte count, hemoglobin, hematocrit, mean corpuscular volume, mean corpuscular hemoglobin, mean corpuscular hemoglobin concentration, anisocytosis index, platelets, mean platelet volume, total leukocyte, lymphocyte, monocyte, and granulocyte levels between the groups tested and the control (Table 3).

The changes in total protein levels (COS5) did not affect the behavior, weight gain or mortality of the animals; thus, the results obtained did not show clinical signs of acute toxicity. As suggested by the OECD, if after the sample administration, in our case with COS1 and COS5, the animals do not show toxicity signs, behavioral changes, or death, the samples can be considered to have low toxicity and be included as category 5, with a lethal dose (LD50) greater than 2000 mg/kg [41]. In view of these results, we decided to carry out the inflammation tests by testing the animals with doses of 30, 300, and 600 mg/kg of COS1 and COS5.

### 2.5. Xylene-Induced Ear Edema Model

The edema formation is one of the main mechanisms involved in the inflammatory process; therefore, the antiedematogenic effect of COS was investigated using the xylene-induced ear edema model. The groups treated by i.g. route at doses of 30, 300, and 600 mg/kg of COS showed significant suppression of edema formation when compared to the group treated with saline by the same route; thus, as shown in Table 4, it was observed that COS1 showed inhibition percentages of 60.81, 89.07, and 77.58% for concentrations of 30, 300, and 600 mg/kg, respectively. Regarding COS5, suppression levels of 81.11, 91.61, and 70.26% were observed for the same concentrations previously mentioned. When compared to the group treated intraperitoneally (i.p.) with dexamethasone (2 mg/kg, 81.11% suppression), these results were shown to be statistically similar.

The xylene-induced ear edema model has often been used to assess the antiedematogenic activity of natural products [42]. Xylene acts as a phlogistic agent, increasing vascular permeability and leading to the edema formation that is characteristic of acute inflammation [43]. This process is related to the action of inflammatory mediators such as histamine, serotonin, acetylcholine, bradykinin, and prostaglandins in the release of neuropeptides, such as substance P, which develops neurogenic inflammation characterized by redness, heat, and edema [44]. Substance P is a neuropeptide with vasodilatory activity that releases NO from endothelial cells, leading to the edema formation through vasodilation and plasma exudation [45]. 

In our study, COS presented results similar to dexamethasone, a potent glucocorticoid that prevents the prostaglandin synthesis through inhibition of phospholipase A2; however, it is not possible to state that the COS mechanism of action is the same as that of glucocorticoids. Studies have shown that COS exhibited inhibitory action on the prostaglandins E2 (PGE2), TNF-α (tumor necrosis factor alpha), and NO production and reduced the enzyme expression important for the inflammatory process, such as cyclooxygenase-2 (COX-2) and induced nitric oxide synthase (iNOS) [15,46]. These factors may be related to the antiflogistic effect of COS; however, further studies are needed to determine their mechanism of action for this model.

### 2.6. Zymosan-Induced Air Pouch Model

**Figure 7:**
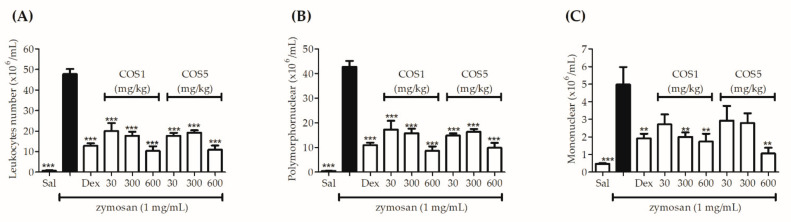
Effects of COS1 and COS 5 in the air pouch model at 30, 300, and 600 mg/kg doses on leukocyte migration. The total leukocyte count (**A**), differential count of the polymorphonuclear cells (**B**), and differential count of the mononuclear cells (**C**). Note: *** *p* < 0.001 and ** *p* < 0.01 compared to the zymosan group (black column). Sal: saline solution (0.9 mg/mL); Dex: dexamethasone (2 mg/kg).

After the acute inflammation induction by zymosan subcutaneous injection (s.c.) in the air pouch, the group treated 30 min before (i.g.) with saline solution showed an intense leukocyte migration to the air pouch; however, the groups treated (i.g.) with COS1 and COS5 at doses of 30, 300, and 600 mg/kg demonstrated significant inhibition of cell migration when compared to the group that received only saline solution (i.g.) as treatment and zymosan (s.c.). As expected, the group treated (i.p.) with dexamethasone significantly reduced the leukocyte migratory effect (73.22%) and was statistically similar to the groups treated with COS1 and COS5 (Figure 7A). 

The differential leukocyte count was performed to assess the migration of the different subpopulations. The data obtained demonstrated that in relation to the zymosan group, all doses of COS1 and COS5 caused a significant reduction in the polymorphonuclear cell migration, and similar inhibition was found in the group treated with dexamethasone (Figure 7B). Mononuclear cells showed reduced migration only at 300 and 600 mg/kg COS1 doses and at the 600 mg/kg COS5 dose (Figure 7C).

**Figure 8:**
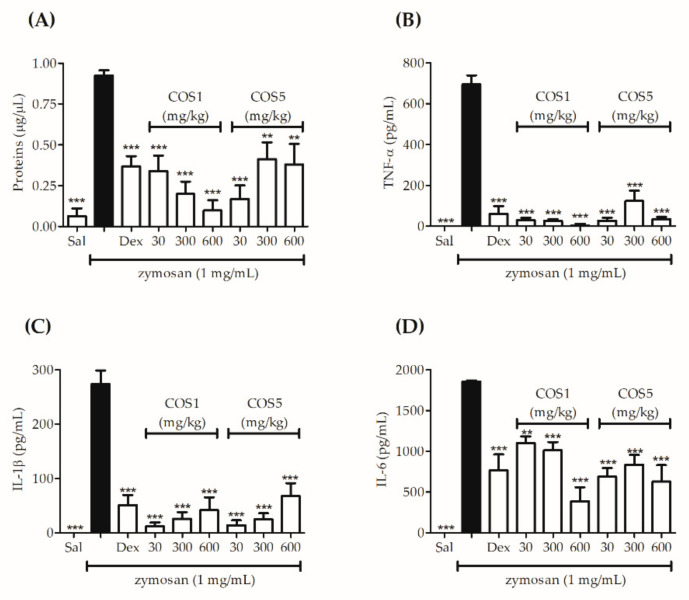
Effects of COS1 and COS5 in the air pouch model at 30, 300, and 600 mg/kg doses on the quantification of total proteins (**A**) and levels of cytokine pro-inflammatory TNF-α (**B**), IL-1β (**C**), and IL-6 (**D**) estimated using ELISA. Note: *** *p* < 0.001 and ** *p* < 0.01 compared to the zymosan group (black column). Sal: saline solution (0.9 mg/mL); Dex: dexamethasone (2 mg/kg).

In addition, the Bradford method was used to quantify the protein plasma extravasation induced by the zymosan administration (s.c.) in the air pouch formed on the animal’s back; thus, the results demonstrated that all doses of COS1 and COS5 significantly reduced the protein concentration in the air pouch when compared to the group that received only saline solution (i.g.) and zymosan (s.c.) (Figure 8A). The cytokine pro-inflammatory TNF-α, IL-1β, and IL-6 levels were also estimated using an ELISA kit. The different doses of COS1 and COS5 significantly reduced (*p* < 0.001) the cytokine levels, similar to the group treated with dexamethasone. 

Another model chosen to assess the anti-inflammatory activity of COS was an air pouch induced by zymosan. The sterile air injection in the mouse’s back leads to the formation of a cavity lined by cells similar to the synovial membrane, resulting in an inflammatory response corresponding to the synovial tissue [47]. Zymosan is a polysaccharide originating from the yeast *Saccharomyces cerevisiae*, which when injected into the air pouch causes a response characterized by plasma exudation and leukocyte migration to the cavity [48]. Studies have demonstrated that the possible zymosan mechanism of action is related to the internalization of this polysaccharide by phagocytic cells, generating an inflammatory response [49]. The recognition of zymosan occurs by Toll-like 2 (TRL-2) and Dectin-1 cell surface receptors present in human neutrophils [48,50]. This interaction induces intracellular processes that result in the adapter protein recruitment, such as MyD88 (myeloid differentiation factor 88), which activates nuclear factor kappa B (NF-kB) transcription, responsible for the pro-inflammatory gene transcription of IL-cytokines 1β, IL-6, and TNF-α [51]. In the literature, it has been shown that chitooligosaccharides have suppressive activity in terms of NF-kB expression and in the production of the cytokines IL-1β, IL-6, and TNF-α [52,53], confirming the results obtained in this study and making it possible that their probable mechanism of action is related to this inflammation pathway; however, as in the xylene-induced ear edema model, further studies are needed to clarify their anti-inflammatory activity.

### 2.7. Healing Test In Vivo

The 30 and 300 mg/kg doses of COS1 and COS5 were demonstrated to have significant effects in the inflammation tests; therefore, they were chosen to be tested in the in vivo healing test.

**Figure 9:**
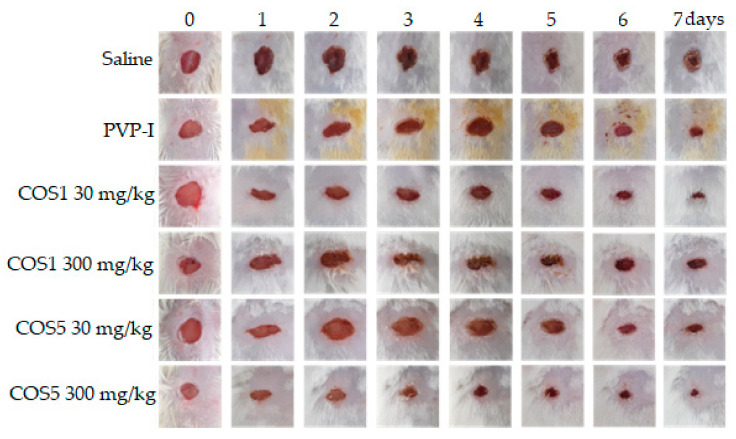
Representative effects of COS1 and COS5 on skin wound healing in vivo.

**Figure 10:**
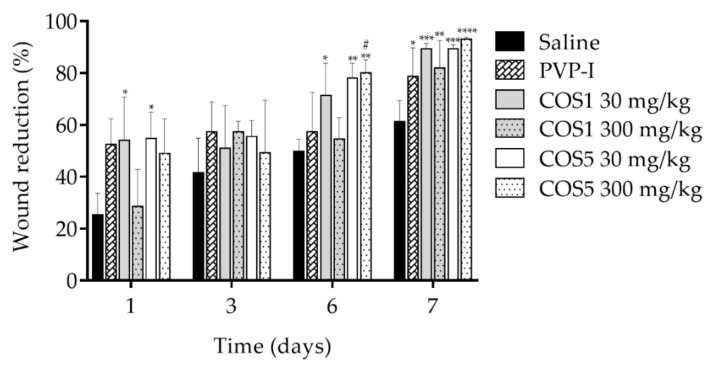
Skin wound extension for days 1, 3, 6, and 7 after local administration of doses of 30 or 300 mg/kg of COS1 or COS5. Note: * p < 0.05, ** p < 0.003, *** p < 0.001, and **** p < 0.0001 compared to the saline group; ^#^ *p* < 0.1 compared to the PVP-I group.

The photodocumentation (Figure 9) demonstrated progress in the healing process in animals treated with COS1 and COS5 during the 7 days of the experiment. The samples treated with COS1 and COS5 exhibited significant reductions in the extent of the lesions when compared to the saline group, especially after the fifth day, revealing similarities with the effects of the positive control povidone–iodine solution (PVP-I). The images of the wound corroborated the results obtained for the extent of the lesion. The group that received the drug of reference showed less wound reduction when compared to the group that received COS5 at 300 mg/kg, especially on the sixth day of the experiment (Figure 10).

The results obtained from this model demonstrated that these molecules have healing activity capable of significantly reducing the wound size when compared to the saline group and showed an average reduction rate around 89.25% ± 2.2 for COS1 30 mg on the last day of the experiment, 82% ± 10.6 for COS1 300 mg, 89.25% ± 1.7 for COS5 30 mg, and 93% ± 0.8 for COS5 300 mg. The saline group showed a reduction of approximately 61.25% ± 8.1 and the group that received PVP-I solution as a positive control showed a reduction of approximately 79% ± 11.0. It was not possible to observe any statistical differences between the groups treated with COS (Figure 10).

Literature data demonstrate that the treatment with COS hydrogel showed a reduction of 84% in the wound size induced in mice, while the group that received only the hydrogel in the absence of the COS decreased by 77% [54]. Although the COS mechanism of action is unknown, some studies indicate that healing action may be related to biological effects such as antimicrobial, anti-inflammatory, and antioxidant activities, as well as the immunostimulant properties acting on the macrophage migration, the NO release, and cytokine release [25]. Macrophages are extremely important in the transition from the inflammatory to the proliferative phase, although the abundance of macrophages and pro-inflammatory cytokines can result in an excessive increase in the permanence in the inflammatory phase, promoting the formation of chronic wounds. Immunomodulatory molecules such as COS are applied to improve the wound healing process [55,56].

In addition, chitosan oligomers incorporated in a nanofiber system (polyvinyl alcohol–silver nanoparticles) were able to activate TGF-β1 [57]. This effect can trigger fibroblast proliferation and migration, making possible the correlation with the results obtained in in vitro tests. The ability of COS to stimulate angiogenesis has also been reported, and as the formation of new vessels aids in the reepithelization and remodeling processes, the action of these molecules may involve shortening the time required for complete wound healing, as noted in the in vivo healing trial [58,59].

The biological effects of COS are influenced by the molecular weight and degree of acetylation and polymerization. Controlling the method used for obtaining oligosaccharides is essential for a better understanding of this relationship [60]; therefore, the method chosen for the COS production was the enzymatic hydrolysis of chitosan by chitosanases from *Baccilus toyonensis*. The hydrolysis times were stipulated in order to investigate the products generated in the degradation, and in this way the results found by other studies of our group pointed to the presence of hexamers in the COS1 sample and hexamers and heptamers in the COS5 sample [18,34]. The presence of these oligomers is related to biocompatibility, as observed in the acute toxicity in vivo and cytotoxicity assays, with their effects on the murine 3T3 fibroblast migration in vitro and with their anti-inflammatory and healing action proven in vivo assays; therefore, due to the promising results achieved with this approach, further investigations are needed to characterize the anti-inflammatory and healing mechanisms of the chitooligosaccharides produced by the chitosanases of *Baccilus toyonensis*. 

## 3. Materials and Methods

### 3.1. Materials

All reagents used in this study were purchased commercially and of adequate analytical grade. Low molecular weight chitosan (deacetylation degree of 85% and molar mass values between 50,000 and 190,000 Da), Bradford reagent and 3-(4,5-dimethyl-thiazol-2-yl)-2,5-diphenyltetrazolium bromide (MTT) were obtained from Sigma-Aldrich Co. (Saint-Louis, MO, USA). The 3T3 cell (ATCC CCL-164) was obtained from American Type Culture Collection (ATCC) (Rockville, MD, USA). DMEM (Dulbecco’s modified Eagle’s medium) supplemented with 10% fetal calf serum and streptomycin (5000 mg/mL)–penicillin (5000 IU) was acquired from GE Healthcare Life Sciences (Logan, UT, USA). The ELISA kit was purchased from e-Biosciences (San Diego, CA, USA) and the biochemical kit from Labtest (Lagoa Santa, MG, Brazil). Bicinchoninic Acid (BCA) was acquired from Thermo Fisher Scientific (Waltham, MA, USA). *B. toyonensis* has a registration number at the National System of Genetic Resource Management and Associated Traditional Knowledge (SisGen) (AD8AE98/Nov 2018). The strain was provided by the Biochemistry Engineering Laboratory from the Federal University of Rio Grande do Norte (UFRN). 

### 3.2. Chitooligosaccharide Production and Characterization

#### 3.2.1. Chitosanase Production and Chitosan Hydrolysis

The chitooligosaccharide production occurred by chitosan enzymatic hydrolysis using chitosanases obtained from *Bacillus toyonensis* CCT 7899. This bacterium was grown in batch mode in a rotary incubator under shaking at 120 rpm and 30 °C for 24 h. The culture medium consisted of peptone (6 g/L), magnesium sulfate (0.5 g/L), dibasic potassium phosphate (1 g/L), and chitosan (2 g/L), with the latter being the main carbon source in order to induce the enzyme synthesis of interest. At the end of the 24 h period, the bacterial culture was transferred to a new liquid medium with an inoculum rate of 10%, which was incubated under the same conditions as before for 36 h. Then, the culture was collected and centrifuged at 4200 rpm for 20 min for cell removal and the sobrenatant, containing the obtained enzyme broth, was stored under refrigeration (–10 °C) until the time of use [61].

To proceed with the hydrolysis process, initially the supernatant obtained previously was thawed at room temperature (25 °C) and was used to determine the enzymatic activity using the 3,5-dinitrosalicylic acid method (DNS) [62]. The total protein measurement was performed using the bicinchoninic acid (BCA) method (Thermo Fisher Scientific, Waltham, MA, USA). The enzyme activity and protein dosage data made it possible to determine the volume of the enzyme broth used in this process.

Afterwards, 4.35 U of chitosanases, equivalent to 5 mg of enzyme extract, was added to the soluble chitosan, which was previously dissolved in HCl (0.1 M), with the pH adjusted to 6.0. Then, the mixture was incubated in a water bath at 55 °C for 1 and 5 min of hydrolysis, generating COS samples named here as COS1 and COS5, respectively. At the end of the hydrolysis time, the reaction was stopped by boiling for 10 min. Then, the samples were centrifuged at 3062× *g* for 20 min, the supernatant was separated to precipitate the COS with absolute alcohol (1:1), and after precipitation the COS samples were concentrated in a vacuum concentrator (Centrivap, Labconco, Kansas, MO, USA) [18]. For the yield calculation, the mass ratio between the COS produced with the chitosan used in the hydrolysis process was considered.

#### 3.2.2. Mass Spectrometry

Regarding the spectrometric process, after the gel filtration process the COS1 samples were named according to the obtained fractions as G1 (fraction 1), G2 (fraction 2), and G3 (fraction 3). Chitosan was also analyzed to compare the chromatographic profiles and to check for the possible presence of reaction products in any of the fractions. All samples were diluted in a mobile phase to prepare a solution with a concentration of 1 mg/mL and filtered through a PTFE filter with a pore diameter of 0.22 µm. The fractions were analyzed by HPLC-MS/MS in positive mode with fragmentation in automatic mode. High-performance liquid chromatography (HPLC) was performed using a chromatograph (Shimadzu model 20A) equipped with a DAD detector, binary pump, oven and automatic injector, and a mass spectrometer (Bruker Daltonics model micrOTOF-Q II) with an ionization source provided by an ESI and QTOF analyzer. The mobile phase consisted of formic acid 0.1% (A) and formic acid in acetonitrile (0.1%, B). The flow used was 1 mL/min, with a LUNA column with phenylhexyl 250 × 4.6 mm × 5 µm in the stationary phase, Phenomenex, an oven temperature 30 °C, and an injection volume of 40 µL. The mobile phase gradient was 2% B during the initial 10 min; from 10 to 30 min it increased from 2% to 100% B; from 30 to 35 min it remained at 100% B; from 35 to 40 min it returned to 2% B; and finally it was maintained at 2% B during the final 5 min. The acquisition range of the DAD detector was 190 to 600 nm. In the mass spectrometer, the drying gas temperature was 220 °C with a flow rate of 9 L/min and a pressure of 4.5 bar. The capillary voltage was 3.5 KV. The MS analysis was performed in scan mode for ions with *m/z* values ranging between 50 and 1500 and MS/MS in the automatic mode, with collision energy varying for each ion. NaTFA was used as an internal and external calibrator [18].

### 3.3. Tests In Vitro

#### 3.3.1. Cytotoxicity Assay (MTT)

The 3T3 cells (ATCC CCL-92) were provided by Hugo A. O. R (Natural Polymer Biotechnology Laboratory, Department of Biochemistry, UFRN, Brazil). The cells were plated in 96-well microplates (5 × 10^3^ cells/well) and kept in Dulbecco’s modified Eagle’s medium (DMEM) (Cultilab, Campinas, SP, Brazil) with serum for adhesion to the surface for 24 h, in an oven at 37 °C with 5% CO_2_ saturation. Subsequently, the culture medium was aspirated and replaced with DMEM without fetal bovine serum, being incubated under the conditions described above. After 24 h, the medium was aspirated and media (100 µL) containing different amounts (100, 250, and 500 µg/mL) of COS1 and COS5 and fetal bovine serum (10%) were added. After 24 h, the cells’ ability to reduce MTT (3-(4,5-dimethylthiazol-2-yl)-2,5-diphenyl-tetrazolium bromide) was determined by adding a solution containing MTT 1 mg/mL in DMEM [63,64]. After 4 h, the supernatant was removed and the formazan crystals were solubilized in ethanol. The plate was maintained under slow stirring for 15 min at room temperature (25 °C) and the absorbance was determined at 570 nm in a microplate spectrophotometer (Epoch, BioTek, Winooski, VT, USA). The results were presented as a percentage of the MTT reduction, considering the negative control absorbance, composed only of the culture (100% of the reduction of MTT). The data are presented as the mean and standard deviations of the three independent experiments carried out in triplicate.

#### 3.3.2. Healing Test (“Scratch”) In Vitro

In a 3T3 murine fibroblast (ATCC CCL-92) cellular confluence in a 24-well plate, a horizontal streak was performed with the aid of a 200 μL tip on the medial surface of each well. To remove debrided cells, the wells were washed with sterile phosphate-buffered saline (PBS; NaCl 137 mM, KCl_3_ mM, KH_2_PO_4_ 1.5 mM, and Na_2_HPO_4_ 10 mM), followed by the addition of COS1 and COS5 at different concentrations (100, 250, and 500 µg/mL) and fetal bovine serum (10%). After the treatment, the wells were monitored using photographs at 0, 6, 12, 18, and 24 h to assess the cell migration towards the free space resulting from the horizontal risk (NIS Elements AR) [65,66].

### 3.4. Tests In Vivo

#### 3.4.1. Animals

Male and female Balb/c (25–30 g) and Swiss (25–35 g) mice from 6 to 8 weeks old were maintained at a temperature of 22 ± 2 °C, with a 12 h light/dark cycle in the sectoral biotherium of the Health Sciences Center of the Federal University of Rio Grande do Norte (UFRN). Each test group was composed of five animals (*n* = 5). After the experiments, the animals were euthanized with xylazine and ketamine (Syntec, Hortolândia, SP, Brazil) at doses of 30 and 300 mg/kg, respectively, via the intraperitoneal route. The experiment was carried out according to the ethics committee guidelines on the use of animals (CEUA) at UFRN, being approved under protocol code 035.041/2017, 4 September 2017.

#### 3.4.2. Acute Toxicity Model

The acute toxicity test was performed according to the Guidelines for the Testing of Chemicals, OECD 423 [41]. The 2000 mg/kg COS1 and COS5 doses were administered by intragastric (i.g.) route to one female mouse following 8 h of fasting. After 48 h, the same dose was administered to four females for a total of five treated animals. The same procedure was performed for the group that received PBS (control group). The animals were observed for 14 days regarding behavioral, neurological, body weight, and mortality changes. At the end of the experiment, blood samples were collected for biochemical and hematological analysis and organs (kidneys, liver, heart, spleen, and lungs) were collected and weighed. The biochemical parameters, including ALT, AST, total protein, albumin, glucose, cholesterol, creatinine, urea, and uric acid, were determined using commercial kits in the automated analyzer Labmax Pleno (Labtest, Lagoa Santa, MG, Brazil). The hematological parameters, including the measured erythrocytes, hemoglobin, hematocrit, mean corpuscular volume (MCV), mean corpuscular hemoglobin (MCH), mean corpuscular hemoglobin concentration (MCHC), anisocytosis index (RDW), platelets, mean platelet volume (MPV), total leukocytes, lymphocytes, monocytes, and granulocytes, were determined using the ABX Micros 60 automated analyzer (Horiba ABX Diagnostics, Kyoto, Japan).

#### 3.4.3. Xylene-Induced Ear Edema Model

Balb/c mice were treated via the i.g. route with 300 μL sterile saline (0.9 mg/mL) or with COS1 and COS5 (30 or 300 or 600 mg/kg). The control group received 100 μL dexamethasone (2 mg/kg) intraperitoneally. Thirty minutes after treatment, the ear edema was induced with 20 μL xylene on the anterior surface and 20 μL on the posterior surface of the right ear. The left ear, which was used as a control, received sterile saline solution (0.9 mg/mL). Then, 15 min after xylene application, the animals were euthanized, both ears were cut into sections of 7 mm using a biopsy punch, then weighed [67]. The edematogenic response was measured as the difference between the weight of the right and left ears, whereby the inhibition level was calculated with the following equation:

$$\mathrm{Edema}\;\mathrm{inhibition}\;\left(\%\right)=1-\frac{\left[\mathrm{Mrt}-\mathrm{Mlt}\right]}{\left[\mathrm{nMrt}-\mathrm{nMlt}\right]}\times 100$$

where “Mrt” corresponds to the arithmetic mean of the right ear weight of animals treated with dexamethasone or COS; “Mlt” corresponds to the arithmetic mean of the left ear weight of animals treated with dexamethasone or COS; “nMrt” refers to the arithmetic mean of the right ear weight of untreated animals; “nMlt” is the arithmetic mean of the left ear weight of untreated animals.

#### 3.4.4. Zymosan-Induced Air Pouch Model

The Swiss mice received 5 mL sterile air on the back via the subcutaneous (s.c.) route to form the pouch. After three days, the bag was reinforced by applying 2.5 mL sterile air in the same place. Six days after the initial pouch formation, the animals were treated by i.g. route with 300 μL sterile saline solution (0.9 mg/mL), COS1, and OQS5 (30, 300, and 600 mg/kg). The control group received 100 μL dexamethasone (2 mg/kg) intraperitoneally. After 30 min, 1 mL zymosan solution (1 mg/mL) was injected into the air pouch. After 6 h, the animals were euthanized, 2 mL sterile saline solution (0.9 mg/kg) was inoculated, and the bag exudate was collected by aspiration with a syringe. The samples were centrifuged at 1500 rpm for 5 min at 4 °C. Here, 1 mL sterile saline solution (0.9 mg/mL) was added to the cell pellet and a total leukocyte count was performed with the aid of the Neubauer chamber, with the sample diluted in the Turk solution (1:10). The results were expressed as the total number of leukocytes (×10^6^/mL) [67,68].

The cell pellet was also used to count leukocyte subpopulations. For this, 20 μL pellet was applied on a slide and then cytocentrifugation (2500 rpm/5 min) was employed (Shandon Cytospin), followed by staining with fast panotic dye (Laborclin, Pinhais, PR, Brazil). The slide analyses were performed under an optical microscope through the oil immersion objective (100× magnification) and 100 cells were counted, differentiating them into mononuclear or polymorphonuclear cell subpopulations. The absolute values are expressed for each subpopulation (×10^6^/mL) [69,70].

The supernatant, described after the centrifugation process, was used for total protein quantification using the Bradford method. In the 96-well plate, 10 μL samples were applied in triplicate. Subsequently, 200 μL Bradford reagent was added and a plate was prepared for 1 min. After 30 min incubation at room temperature (25 °C), the microplate reader (Epoch, BioTek) was read at a wavelength of 595 nm [71,72]. The supernatant exudates from the air pouch were also used to measure cytokine levels (TNF-α, IL-1β, and IL-6). The assay was performed using an ELISA Kit (e-Biosciences, San Diego, CA, USA) according to the manufacturer’s instructions.

#### 3.4.5. Wound Induction

The animals were anesthetized with ketamine 10% and xylazine 2% for surgical wound induction. The trichotomy and antisepsis with 70% alcohol were performed on the animal’s dorsal region followed by the wound skin induction with the aid of a punch (5 mm) in that region. The animals were topically treated with daily 100 μL sterile saline, povidone-iodine solution (PVP-I), COS1, or COS5 (30 and 300 mg/kg) for 7 days. Photo documentation using the ImageJ 1.52a program software (National Institutes of Health, MD, USA) was performed daily to assess tissue repair and the lesion extent was measured from the first to the seventh day [73,74]. The size on the initial day of the experiment was considered as 100% (0% reduction). At the end of the experiment the animals were euthanized. 

### 3.5. Statistical Analysis

Values are expressed as means ± standard of the mean (SEM). Statistical analyses were performed by ANOVA, followed by the Tukey’s test using GraphPad Prism^®^ version 7.00 (GraphPad software, San Diego, CA, USA). Differences in the mean values of * p < 0.05, ** p < 0.003, *** p < 0.001, and **** p < 0.0001 were considered as statistically significant.

## 4. Conclusions

According to the obtained results, this study revealed that chitooligosaccharides produced through chitosan hydrolysis by chitosanases from *Baccilus toyonensis* have oligomer predominance and healing and anti-inflammatory actions. The substances’ biocompatibility in vitro and in vivo was reasserted. The ability to induce 3T3 fibroblast migration was evidenced for the first time, highlighting that COS5 (500 µg/mL) completely closed the slit in cell culture after 12 h of experiment. On the sixth day of the in vivo healing trial, greater wound reduction was noted in the group treated with COS5 (300 mg/kg) when compared to the PVP-I group. The inflammatory tests showed significant antiedematogenic responses, reduced protein exudate, and decreased cytokine production and leukocyte migration for COS1 and COS5. As such, COS have the potential to be used in the treatment of dermatological disorders; however, the tests carried out did not exhaust the questions related to the anti-inflammatory and healing actions of COS, so additional studies are needed to elucidate the mechanisms of action of these molecules.

## Figures and Tables

**Table 1:** Body weight gain and relative organ weight of mice treated by intragastric route with COS1 and COS5 in the acute toxicity model.

Body Weight Gain	PBS	COS 1	COS 5
Initial weight (g)	29.550 ± 0.610	29.500 ± 0.431 ^ns^	29.310 ± 0.588 ^ns^
Final weight (g)	33.390 ± 1.002	33.040 ± 0.716 ^ns^	32.920 ± 0.643 ^ns^
Body weight gain (%)	11.39 ± 0.944	10.63 ± 1.488 ^ns^	10.92 ± 1.744 ^ns^
**Relative organ weight** **(g/10 g of body)**			
Lungs	0.053 ± 0.003	0.057 ± 0.003 ^ns^	0.062 ± 0.003 *
Heart	0.085 ± 0.008	0.076 ± 0.006 ^ns^	0.071 ± 0.004 ^ns^
Liver	0.542 ± 0.020	0.500 ± 0.010 ^ns^	0.508 ± 0.023 ^ns^
Spleen	0.056 ± 0.003	0.052 ± 0.008 ^ns^	0.061 ± 0.007 ^ns^
Kidneys	0.125 ± 0.005	0.122 ± 0.005 ^ns^	0.123 ± 0.009 ^ns^

Data shown as means ± SEM (*n* = 5). Note: * *p* < 0.001 compared with PBS group. ^ns^ Indicates no significant difference compared with PBS group. PBS: phosphate-buffered saline. COS1 and COS5, 2000 mg/kg.

**Table 2:** Biochemical parameters of mice treated by intragastric route with COS1 and COS5 in acute an toxicity model.

Biochemical Parameters	PBS	COS1	COS5
ALT (U/L)	55.20 ± 1.855	62.80 ± 2.245 ^ns^	60.50 ± 1.360 ^ns^
AST (U/L)	105.20 ± 4.434	94.50 ± 2.245 ^ns^	106.60 ± 4.032 ^ns^
Total protein (g/dL)	5.88 ± 0.558	5.92 ± 0.066 ^ns^	6.32 ± 0.102 **
Albumin (g/dL)	1.85 ± 0.028	1.82 ± 0.020 ^ns^	1.82 ± 0.047 ^ns^
Glucose (mg/dL)	83.40 ± 5.036	84.60 ± 4.173 ^ns^	83.70 ± 3.675 ^ns^
Cholesterol (mg/dL)	90.00 ± 4.868	104.20 ± 6.256 ^ns^	102.60 ± 4.411 ^ns^
Creatinine (mg/dL)	0.18 ± 0.004	0.17 ± 0.013 ^ns^	0.20 ± 0.023 ^ns^
Urea (mg/dL)	33.00 ± 3.342	35.50 ± 0.9574 ^ns^	32.00 ± 2.214 ^ns^
Acid uric (U/L)	1.32 ± 0.126	1.45 ± 0.125 ^ns^	1.42 ± 0.197 ^ns^

Data shown as means ± SEM (*n* = 5). Note: ** *p* < 0.01 compared with PBS group. ^ns^ Indicates no significant difference compared with PBS group. PBS: phosphate-buffered saline; ALT: alanine aminotransferase; AST: aspartate aminotransferase. COS1 and COS5, 2000 mg/kg.

**Table 3:** Hematological parameters of mice treated by intragastric route with COS1 and COS5 in an acute toxicity model.

Hematological Parameters	PBS	COS1	COS5
Erythrocytes (10^6^/mm^3^)	8.02 ± 0.300	7.58 ± 0.537 ^ns^	8.85 ± 0.279 ^ns^
Hemoglobin (g/dL)	18.08 ± 0.759	16.62 ± 1.175 ^ns^	18.24 ± 0.201 ^ns^
Hematocrit (%)	45.22 ± 2.364	41.24 ± 3.88 ^ns^	50.04 ± 1.825 ^ns^
MCV (fL)	56.20 ± 1.319	54.00 ± 1.673 ^ns^	55.20 ± 0.374 ^ns^
MCH (pg)	22.54 ± 0.177	21.96 ± 0.668 ^ns^	20.72 ± 0.532 ^ns^
MCHC (g/dL)	40.18 ± 0.774	40.78 ± 1.405 ^ns^	38.40 ± 0.418 ^ns^
RDW (%)	15.98 ± 0.504	15.92 ± 0.457 ^ns^	15.88 ± 0.096 ^ns^
Platelets (10^3^/mm^3^)	617.6 ± 1.79	655.2 ± 2.98 ^ns^	576.4 ± 1.546 ^ns^
MPV (fL)	7.00 ± 0.151	7.30 ± 0.202 ^ns^	7.58 ± 0.576 ^ns^
Leukocytes (10^3^/mm^3^)	6.26 ± 0.728	7.22 ± 1.304 ^ns^	5.90 ± 0.862 ^ns^
Lymphocytes (10^3^/mm^3^)	5.17 ± 0.773	6.16 ± 1.127 ^ns^	4.94 ± 0.681 ^ns^
Monocytes (10^3^/mm^3^)	0.52 ± 0.058	0.66 ± 0.074 ^ns^	0.42 ± 0.048 ^ns^
**Granulocytes (10^3^/mm^3^)**	0.38 ± 0.073	0.40 ± 0.083 ^ns^	0.44 ± 0.092 ^ns^

Data shown as means ± SEM (*n* = 5). ^ns^ Indicates no significant difference compared with PBS group. PBS: phosphate-buffered saline; MCV: mean corpuscular volume; MCH: mean corpuscular hemoglobin; MCHC: mean corpuscular hemoglobin concentration; RDW: anisocytosis index; MPV: mean platelet volume. COS1 and COS5, 2000 mg/kg.

**Table 4:** Antiedematogenic effects of chitooligosaccharides (COS1 and COS5) after 1 and 5 min of hydrolysis in the xylene-induced ear edema model.

Groups	Dose (mg/kg)	Difference (mg)	Inhibition (%)
Saline Dexamethasone	---- 2	28.380 ± 4.547 5.360 ± 0.980 ***	---- 81.11
COS1	30	11.12 ± 1.192 ***	60.81
COS1	300	3.100 ± 2.691 ***	89.07
COS1	600	6.360 ± 1.589 ***	77.58
COS5	30	5.360 ± 2.654 ***	81.11
COS5	300	2.380 ± 2.216 ***	91.61
COS5	600	8.440 ± 2.758 ***	70.26

Values are means ± standard deviation (*n* = 5). Note: *** p < 0.001 compared with the group treated with saline.

## Data Availability

Not applicable.
